# DFT/TDDFT calculations of geometry optimization, electronic structure and spectral properties of clevudine and telbivudine for treatment of chronic hepatitis B

**DOI:** 10.1038/s41598-024-58599-2

**Published:** 2024-04-08

**Authors:** Dereje Fedasa Tegegn, Habtamu Zewude Belachew, Ayodeji Olalekan Salau

**Affiliations:** 1 Department of Chemistry, College of Natural and Computational Science, Dambi Dollo University, Dambi Dollo, P. O. Box. 260, Oromia, Ethiopia; 2 Department of Electrical/Electronics and Computer Engineering, Afe Babalola University, Ado-Ekiti, Nigeria; 3 Saveetha School of Engineering, Saveetha Institute of Medical and Technical Sciences, Chennai, Tamil Nadu India

**Keywords:** Density functional theory, Electronic structure, Clevudine, Telbivudine, Biochemistry, Biological techniques, Biotechnology

## Abstract

Chronic hepatitis B remains a worldwide health concern. Presently, many drugs, such as Clevudine and Telbivudine, are recommended for the treatment of chronic hepatitis B disease. For this purpose, the quantum chemical analysis of E_LUMO-HOMO_ (E_gap_), ionization potential (IP), electron affinity (EA), electronegativity (EN), chemical hardness (η), chemical potential (μ), chemical softness (S), electrophilicity index (ω), electron accepting capability (ω^+^), electron-donating capability (ω^-^), Nucleophilicity index (N), additional electronic charge (∆N_max_), Optical softness (σ^0^) and Dipole Moment, IR and UV–Vis spectra, molecular electrostatic potential (MEP) profile, Mulliken charge analysis, natural bond orbital (NBO) were examined in this study. The dipole moment of the compounds suggests their binding pose and predicted binding affinity. The electrophilic and nucleophilic regions were identified, and techniques such as NBO, UV–Vis, and IR were used to gain insights into the molecular structure, electronic transitions, and potential drug design for Hepatitis B treatment. Calculations for this study were carried out using the Gaussian 09 program package coupled with the DFT/TDDFT technique. The hybrid B3LYP functional method and the 6-311++G(d, p) basis set were used for the calculations.

## Introduction

Hepatitis B virus is a kind of Hepadnaviridae virus with partly double-stranded circular DNA^1,2^. This virus causes liver cancer and cirrhosis, which affects around 350 million people and causes 1 million deaths each year^3^. A substantial improvement in oral nucleoside derivatives has been made to treat hepatitis B. Therefore, the recently ratified drugs for the treatment of chronic hepatitis B disease are clevudine and telbivudine. Among them, the status of clevudine has been studied as an antiviral drug in the context of the continuing progress of fluorine-containing compounds^4^. Telbivudine was approved based on randomized studies compared to other recognized Hepatitis B agents^5^. However, there are no adequate studies on Hepatitis B infection among people, especially in developing countries, including Ethiopia^6,7^.

Clevudine is a synthetic pyrimidine nucleoside derivative that hinders hepatitis B virus DNA synthesis^8^. It has the chemical formula 1-(2S, 3R, 4S, 5S).-3-fluoro-4-hydroxy-5-(hydroxymethyl)oxolan-2-yl)-5-methylpyrimidine-2,4-dione. Telbivudine is a synthetic thymidine nucleoside analog that is an unmodified β-L enantiomer with activity against HBV^9^. Cellular kinases phosphorylate it to its triphosphate form, which can inhibit HBV DNA polymerase by competing with thymidine 5′-triphosphate^10,11^. It has the chemical formula 1-(2R,4R,5S).-4-hydroxy-5-(hydroxymethyl)oxolan-2-yl)-5-methylpyrimidine-2,4-dione.

**Figure 1:**
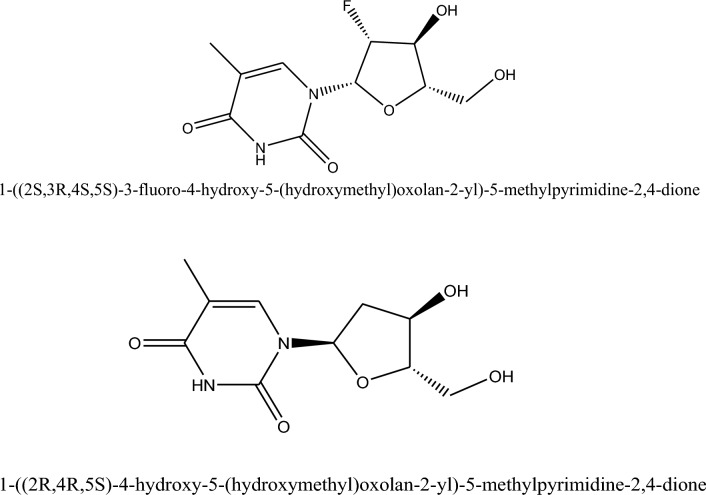
Clevudine (1-((2S,3R,4S,5S)-3-fluoro-4-hydroxy-5-(hydroxymethyl)oxolan-2-yl)-5-methylpyrimidine-2,4-dione) and Telbivudine (1-(2R,4R,5S)-4-hydroxy-5-(hydroxymethyl)oxolan-2-yl)-5-methylpyrimidine-2,4-dione).

The most common and widely used theoretical method for computing the electronic structural features of atoms and large molecules is the density functional theory. It is an important method to study the electronic properties of different isolated drug molecules, the direction of drug delivery systems, and the way drug-receptor interactions occur. The energetic properties of drug molecules are also of significant interest in a drug design. Ionization energy, relative energy, electron affinity, and metal–ligand bond strength can be effectively studied through DFT. The foundation of any computational drug study is the prediction of the lowest energy conformation of that drug molecule^12^. Figure 1 shows the clevudine and telbivudine structures.

Therefore, it is possible to predict drug-receptor interaction, and spectral and energetic properties of clevudine and telbivudine by DFT. Polyamidoamine dendrimer molecular interactions that suppress the growth of the hepatitis virus have been theoretically studied^13^. The molecular structure, electronic properties, spectral properties (IR and UV–Vis), and any quantum mechanical calculations of clevudine and telbivudine have not been reported in the literature review. In this study, the molecular and electronic properties, spectral properties, and some quantum mechanical calculations of clevudine and telbivudine were studied. The aim of this study is a computational or theoretical analysis of the geometry, electronic structure, and spectral properties of clevudine and telbivudine for the treatment of Hepatitis B.

## Method

### Proposed computational method

Density functional theory (DFT) gives precise and trustworthy information about the compounds’ shape, rotational barrier, vibrational frequency, and electronic properties^14,15^. Calculations for this investigation were carried out utilizing the Gaussian 09 program package coupled with the TD-DFT technique. The hybrid B3LYP functional method and the 6-311++G(d, p) basis set were used for the calculations^28^. B3LYP is one of the most popular and successful functionals for drug design because it can balance the accuracy and efficiency of the calculations. B3LYP can reproduce the geometry, vibrational frequencies, dipole moments, and reaction energies of many drug molecules. B3LYP can also capture the electronic and magnetic properties of drug molecules, such as ionization potentials, electron affinities, spin densities, and NMR chemical shifts. B3LYP has been widely adopted and considered a workhorse functional in quantum chemistry. Its popularity stems from its balance between accuracy and computational cost. Researchers often choose it because it provides reasonable results across a broad range of systems, including organic and inorganic compounds^16^.

The geometry of the minimum energy conformers was optimized with no constraints. The optimized structures of clevudine and telbivudine were confirmed as true minima through frequency calculations at the same level of theory (lack of any imaginary frequency). The bond parameters (bond length, bond angle, and dihedral angle) of both Clevudine and Telbivudine were taken from the optimized geometry. The electronic structure and global reactivity descriptor parameters such as the ionization potential (IP), electron affinity (EA), electronegativity (χ), chemical hardness (η), chemical potential (μ), chemical softness (S), electrophilicity index (ω), electron accepting capability (ω^+^), electron donor capability (ω^-^), nucleophilicity index (N), additional electronic charge (∆N_max_), optical softness (σ^0^) and dipole moment were calculated from optimized structure Clevudine and Telbivudine compounds.

Excited state calculations were achieved by time-dependent density functional theory at the B3LYP/6-311++G(d, p) level basis set. The UV–visible absorption spectra of clevudine and telbivudine drugs were achieved in the gaseous phase, methanol and water solvents. Water and methanol was used as a solvent, and the solution calculations were carried out using the Polarizable Continuum Model (PCM), as well as the integral equation formalism variant (IEF-PCM), as implemented in Gaussian 09. Calculating the geometries and the conceptual DFT indices in the gas phase is important for therapeutical drugs because it allows to evaluate their chemical reactivity, stability, and interactions with other molecules. Conceptual DFT is a branch of density functional theory that uses response functions, such as the Fukui function, to describe the tendency of a molecule to donate or accept electrons during a chemical reaction^22^. These functions can be derived from the molecular geometry and the electron density, which are usually computed in the gas phase as a first approximation. By knowing the geometries and the conceptual DFT indices of therapeutical drugs, one can predict their pharmacological properties, such as binding affinity, solvation energy, and bioavailability^23^. This can help to design more effective and safer drugs for various diseases. The findings of UV–Visible and IR data produced from the Gaussian 09 program package are simulated using the Gabedit software. From the HOMO and LUMO orbital energies, the frontier molecular orbitals (FMOs) and energy gaps are explored. Clevudine and Telbivudine's Mulliken atomic charges were calculated using the optimized geometry. The Natural Bond Orbital (NBO) study was calculated using the Gaussian 09 software suite. It is a suitable criterion to provide relevant data on intramolecular and intermolecular interactions to analyze the charge transfer in a system between donor and acceptor NBOs. We used molecular electrostatic potential (MEP) plots to locate the reactive site for electrophilic and nucleophilic attacks^24^.

## Results and discussion

### Molecular geometry

**Figure 2:**
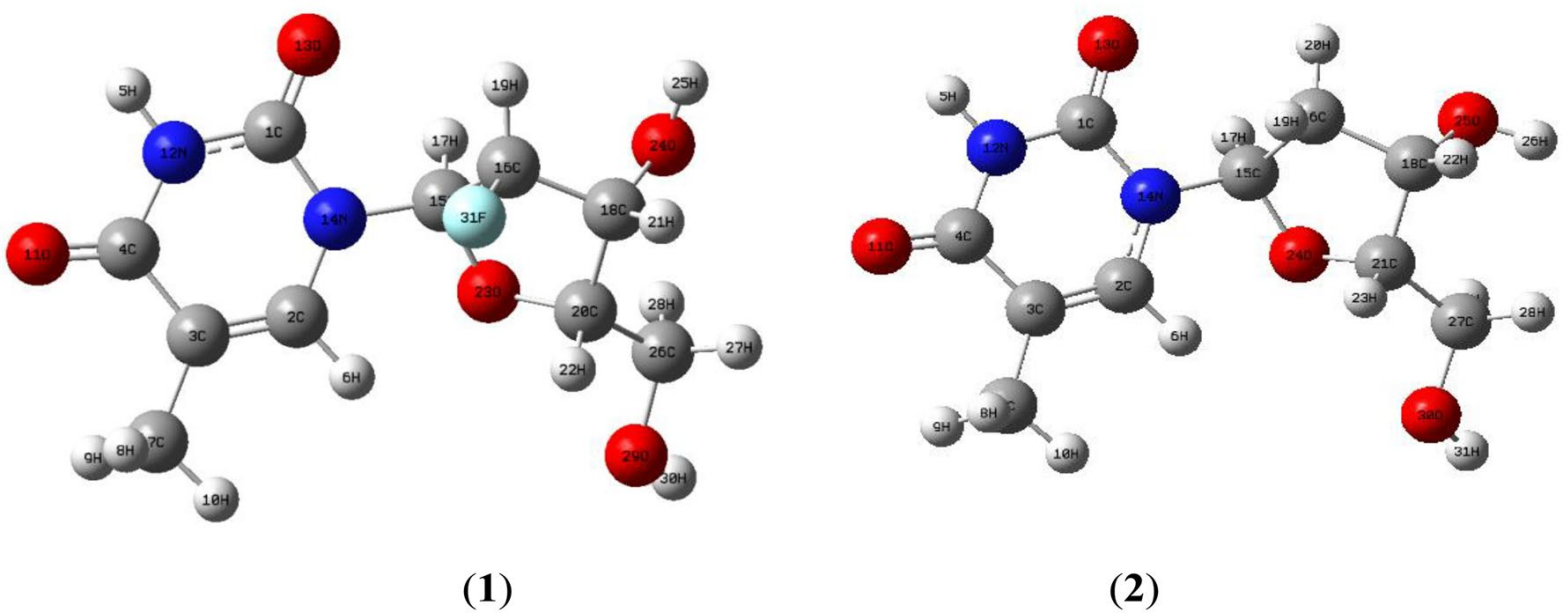
The optimized geometries of Clevudine (**1**) and Telbivudine (**2**) compounds were determined at B3LYP/6-311++G(d p) level in the ground state.

The structural bond characteristics of clevudine and telbivudine are geometrically optimized in their ground states. As determined using B3LYP/6-311++G(d,p) levels, the optimal bond lengths, bond angles, and dihedral angles of both Clevudine and Telbuvidine are reported in Table 1, and the optimized geometries are displayed in Fig. 2. According to the theoretical calculations, clevudine and telbivudine have a non-planar structure with C_1_ point group symmetry. The carbon–carbon bond lengths in the benzene ring of compounds Clevudine and Telbuvidine range between 1.351 and 1.352 Å, which support the double-bond character, and 1.460 Å bond lengths supports the single bond character, respectively. Bond order and bond strength are connected. Shorter bond lengths are associated with higher bond order values, and vice versa. According to the bond order analyses, the weakest bonds cleave preferentially and may have a low pi-bond nature. Table 1 shows that the bonds between C1-N12 have a low pi-bond character, with bond order values of 1.378 Å and 1.381 Å for compounds Clevudine and Telbuvidine, respectively. The C4-O11 and C1-O13 bond order values are in the range 1.219 Å which describes the double bond character, while the O29-H30 and O24-H25 bond order values are approximately the same, which shows the single-bond character.

**Table 1:** The bond parameters of Clevudine (**1**) and Telbivudine (**2**) compounds.

Bond parameters					
Bond length (Å)					
	**1**	**2**		**1**	**2**
R(C1-N12)	1.378	1.381	R(C15-H17)	1.091	1.089
R(C1-O13)	1.220	1.219	R(C15-O23)	1.410	1.407
R(C1-N14)	1.389	1.387	R(C16-C18)	1.531	1.528
R(C2-C3)	1.351	1.352	R(C16-H19)	1.089	1.091
R(C2-H6)	1.080	1.081	R(C16-F31)	1.399	–
R(C2-N14)	1.382	1.380	R(C18-C20)	1.531	1.536
R(C3-C4)	1.460	1.460	R(C18-H21)	1.094	1.095
R(C3-C7)	1.501	1.501	R(C18-O24)	1.424	1.428
R(C4-O11)	1.218	1.219	R(C20-H22)	1.096	1.099
R(C4-N12)	1.407	1.406	R(C20-O23)	1.449	1.441
R(H5-N12)	1.012	1.012	R(C20-C26)	1.514	1.513
R(C7-H8)	1.093	1.093	R(O24-H25)	0.962	0.962
R(C7-H9)	1.093	1.093	R(C26-H27)	1.098	1.100
R(C7-H10)	1.091	1.091	R(C26-H28)	1.093	1.094
R(N14-C15)	1.471	1.496	R(C26-O29)	1.425	1.425
R(C15-C16)	1.540	1.544	R(O29-H30)	0.961	0.961

Bond angle (^o^)					
	**1**	**2**		**1**	**2**
A(N12-C1-O13)	123.48	123.28	A(C16-C15-H17)	109.13	112.11
A(N12-C1-N14)	113.82	113.92	A(C16-C15-O23)	106.37	107.03
A(O13-C1-N14)	122.68	122.78	A(H17-C15-O23)	110.56	108.87
A(C3-C2-H6)	122.47	122.22	A(C15-C16-C18)	101.96	103.91
A(C3-C2-N14)	123.47	123.72	A(C15-C16-H19)	112.90	110.11
A(H6-C2-N14)	114.03	114.03	A(C15-C16-F31)	109.97	112.12
A(C2-C3-C4)	118.52	118.45	A(C18-C16-H19)	115.25	110.32
A(C2-C3-C7)	123.27	123.30	A(C18-C16-F31)	108.44	101.49
A(C4-C3-C7)	118.20	118.22	A(H19-C16-F31)	108.10	112.06
A(C3-C4-O11)	125.85	125.83	A(C16-C18-C20)	101.25	111.94
A(C3-C4-N12)	114.03	113.92	A(C16-C18-H21)	111.54	101.49
A(O11-C4-N12)	120.11	120.23	A(C16-C18-O24)	110.71	112.06
A(C3-C7-H8)	110.84	110.90	A(C20-C18-H21)	111.87	113.23
A(C3-C7-H9)	110.88	110.88	A(C20-C18-O24)	109.02	110.46
A(C3-C7-H10)	110.96	110.99	A(H21-C18-O24)	111.93	108.90
A(H8-C7-H9)	106.69	106.61	A(C18-C20-H22)	108.85	108.90
A(H8-C7-H10)	108.65	108.66	A(C18-C20-O23)	105.31	104.47
A(H9-C7-H10)	108.66	108.64	A(C18-C20-C26)	115.36	116.46
A(C1-N12-C4)	128.10	128.16	A(H22-C20-O23)	108.28	109.55
A(C1-N12-H5)	115.50	115.44	A(H22-C20-C26)	108.62	108.16
A(C4-N12-H5)	116.34	116.39	A(O23-C20-C26)	110.17	109.12
A(C1-N14-C2)	121.97	121.76	A(C15-O23-C20)	110.62	110.236
A(C1-N14-C15)	115.86	115.89	A(C18-O24-H25)	109.33	109.57
A(C2-N14-C15)	122.08	122.25	A(C20-C26-H27)	108.64	108.85
A(N14-C15-C16)	113.61	112.87	A(C20-C26-H28)	109.32	108.98
A(N14-C15-H17)	107.11	106.43	A(C20-C26-O29)	107.54	108.13
A(N14-C15-O23)	110.06	109.45	A(C26-O29-H30)	108.96	108.94

Dihedral angle (*ϕ*)					
	**1**	**2**		**1**	**2**
D(O13-C1-N12-C4)	− 177.05	179.58	D(C3-C2-N14-C15)	178.31	− 178.81
D(O13-C1-N12-H5)	0.3158	0.0221	D(C2-C3-C4-O11)	179.88	− 179.89
D(N14-C1-N12-C4)	2.7525	− 0.708	D(C2-C3-C7-H8)	120.83	121.07
D(N14-C1-N12-H5)	− 179.87	179.72	D(N14-C15-O23-C20)	− 132.03	134.56
D(N12-C1-N14-C2)	− 2.7455	2.0657	D(C16-C15-O23-C20)	− 8.5443	16.422
D(N12-C1-N14-C15)	− 179.59	178.79	D(H17-C15-O23-C20)	109.82	− 104.34
D(O13-C1-N14-C2)	177.06	− 178.22	D(C15-C16-C18-C20)	− 36.956	15.841
D(O13-C1-N14-C15)	0.2139	− 1.4999	D(C20-C18-O24-H25)	− 176.52	− 152.49
D(H6-C2-C3-C4)	− 178.50	− 178.17	D(H21-C18-O24-H25)	− 52.214	− 81.221
D(N14-C2-C3-C7)	179.43	− 179.91	D(C20-C26-O29-H30)	166.60	163.28

### Molecular orbital properties and global reactivity descriptors

**Figure 3:**
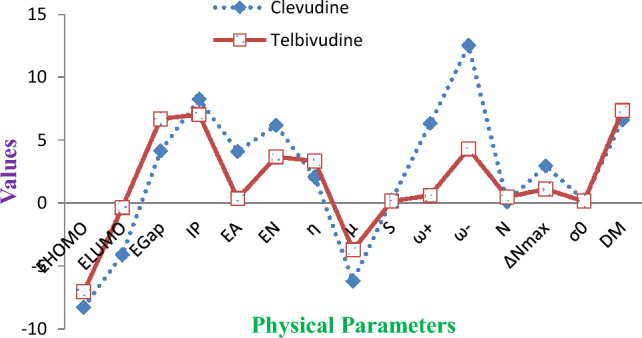
Chemical reactivity descriptors of Clevudine and Telbivudine.

**Figure 4:**
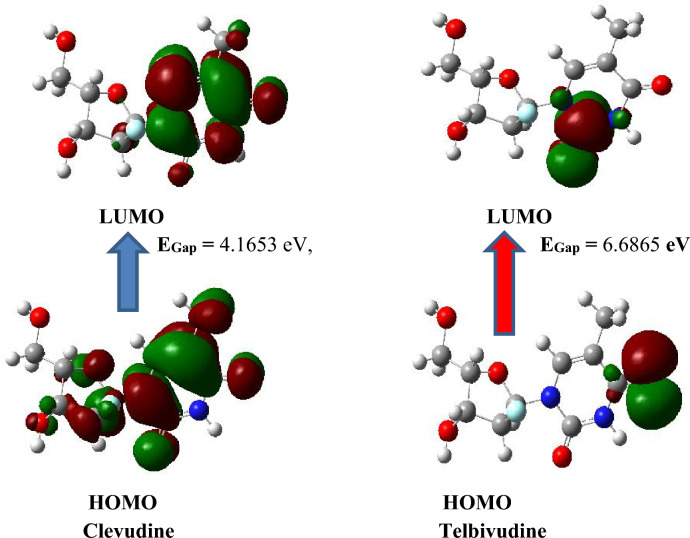
The HOMO and LUMO 3D graphs for the compounds clevudine and telbivudine.

The Frontier Molecular Orbitals (FMO) regulates the way that medications interact with their receptors and provide precise qualitative proof of the electrons’ susceptibility. The LUMO is the lowest energy orbital with a place for electrons to enter, making it an electron acceptor^17^. The HOMO is the highest energy orbital occupied by electrons, making it an electron donor. Moreover, HOMO and LUMO are very significant quantum chemical parameters to predict the reactivity of the compounds^25–27^. They are used to study significant chemical reactivity descriptors, including ionization potential (IP), electron affinity (EA), electronegativity (EN), chemical hardness (η), chemical potential (μ), chemical softness (S), electrophilicity index (ω), electron-accepting capability (ω+), electron-donating capability (ω−), nucleophilicity index (N), additional electronic charge (∆Nmax), optical softness (σo) and dipole moment (DM)^18^. Clevudine and Telbivudine's chemical reactivity descriptors are provided in Fig. 3, and their HOMO and LUMO plots are shown in Fig. 4, respectively. By using the DFT approach and the B3LYP/6-311G++(d,p) basis set, the energies of the HOMOs and LUMOs of all global reactivity descriptors of clevudine and telbivudine compounds were determined. The results are presented in Table 2. The collected data reveals that the energy gaps of Clevudine and Telbivudine are 4.1653 eV and 6.6865 eV, respectively. The increasing order of Egap is 1 $<$ 2. Telbivudine has a larger energy gap than clevudine. Because it is more polarizable, frequently exhibits high chemical reactivity, and has a low level of kinetic stability, a soft molecule has a small gap energy. As evident in Table 2, Clevudine has the highest IP (8.2629 eV), EA (4.0976 eV), S (0.2401 eV), ω^+^ (6.3401 eV), ω^−^ (12.5203 eV), ω (9.1696 eV), and ∆N_max_ (2.9673 eV). Telbivudine has the highest η (3.3435 eV), μ (− 3.6986 eV), N (0.4888 eV), and dipole moment (7.3550 eV). The dipole moment of the compounds under study is in the order of Clevudine < Telbivudine, according to DFT calculated data. A specific target protein's high dipole moment could reveal its binding position and the fulfillment of the predicted binding affinity.

**Table 2:** Calculated energy and chemical reactivity descriptors of compounds 1 and 2.

S. No.	Physical parameters	**1**	**2**
1	E_HOMO_ (Hartree)	− 0.30367	− 0.25881
2	E_LUMO_ (Hartree)	− 0.15059	− 0.01305
3	E_HOMO_ (eV)	− 8.2629	− 7.0422
4	E_LUMO_ (eV)	− 4.0976	− 0.3551
5	E_Gap_ (eV)	4.1653	6.6865
6	Ionization potentials, IP (eV)	8.2629	7.0422
7	Electron affinity, EA (eV)	4.0976	0.3551
8	Electronegativity, EN (eV)	6.1802	3.6986
9	Chemical hardness, *η* (eV)	2.0827	3.3435
10	Chemical potential, *μ* (eV)	− 6.1802	− 3.6986
11	Chemical softness, *S* (eV^−1^)	0.2401	0.1495
12	Electrophilicity index, *ω* (eV)	9.1696	2.0457
13	Electron accepting capability (*ω*^+^)	6.3401	0.6143
14	Electron donating capability (*ω*^*−*^)	12.5203	4.3130
15	Nucleophilicity index (N)	0.1091	0.4888
16	Additional electronic charge (∆N_max_)	2.9673	1.1062
17	Optical softness (σ^0^)	0.2401	0.1495
18	Dipole moment (Debye)	6.6060	7.3550

|∆*E*|= E_LUMO_−E_HOMO_, IP = −E_HOMO_, EA = −E_LUMO_, *EN* = $$\frac{{\left( {I + A} \right)}}{2}$$, *η* = =  $$\frac{{\left( {I - A} \right)}}{2}$$, *μ* = − $$\frac{{\left( {I + A} \right)}}{2}$$, *S* = $$\frac{1}{{\left( {2\eta } \right)}},$$ *ω* = $$\frac{{\mu^{2} }}{{\left( {2\eta } \right)}}$$*, ω*^+^  = $$\frac{{\left( {I + 3A} \right)^{2} }}{{16\left( {I - A} \right)}}$$*, ω*^*−*^=  = $$\frac{{\left( {3I + A} \right)^{2} }}{{16\left( {I - A} \right) }}, $$ *N* =  = $$\frac{1}{\omega }$$*,* ∆N_max_ = $$\frac{ - \mu }{{\eta }}$$*, and* σ^o^ = $$\frac{1}{\Delta E }$$.

### Molecular electrostatic potential (MEP)

**Figure 5:**
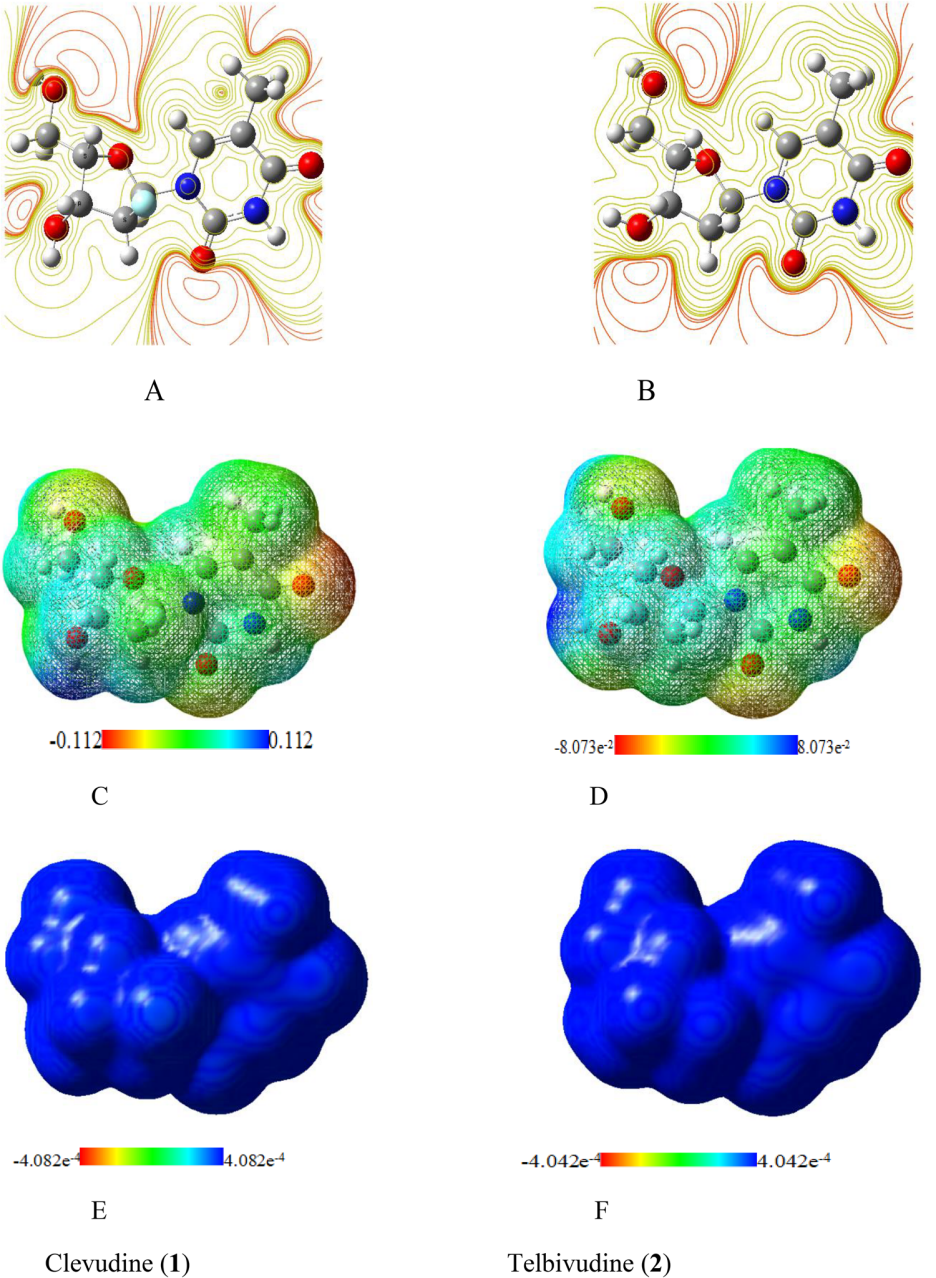
Electron density from total SCF density: (**A**) and (**B**) electron density from contour isosurface density: (**C**) and (**D**); electron density from Alpha SCF density: (**E**) and (**F**) surfaces around both molecules.

It offers details on the size and shape of the molecules that make up the positive, negative, and neutral electrostatic potentials^29^. Additionally, the MEP is a useful tool for predicting how the drugs will react to electrophilic and nucleophilic assaults^19,41–45^. Figure 5 illustrates the computation of the MEP for the tested clevudine and telbivudine compounds using the same method and identical basis sets. The color red, which represents the highest negative region in the MEP, serves as an example of the preferred site for electrophilic assault. An attacking electrophile is thereby drawn positively to the blue regions and negatively to the negatively charged sites. It is apparent that the type of atoms and their electrical nature affected the size, shape, and orientation of molecules as well as the orientation of the negative, positive, and neutral electrostatic potential depending on the compounds. Variations in how the electrostatic potential around the molecules is mapped may be what principally contribute to the variance in their binding affinity to the active site receptor. The locations of the studied compounds’ likely interactions are shown on MEP surfaces. Areas that are vulnerable to electrophilic and nucleophilic attacks are indicated by the red and blue colors, respectively. Clevudine and telbivudine compounds have an electrophilic and nucleophilic region that is the C=O group on the pyrimidine ring. In increasing sequence, the following electron density values rise Red > Orange > Yellow > Green > Blue.

### Mulliken atomic charges

**Figure 6:**
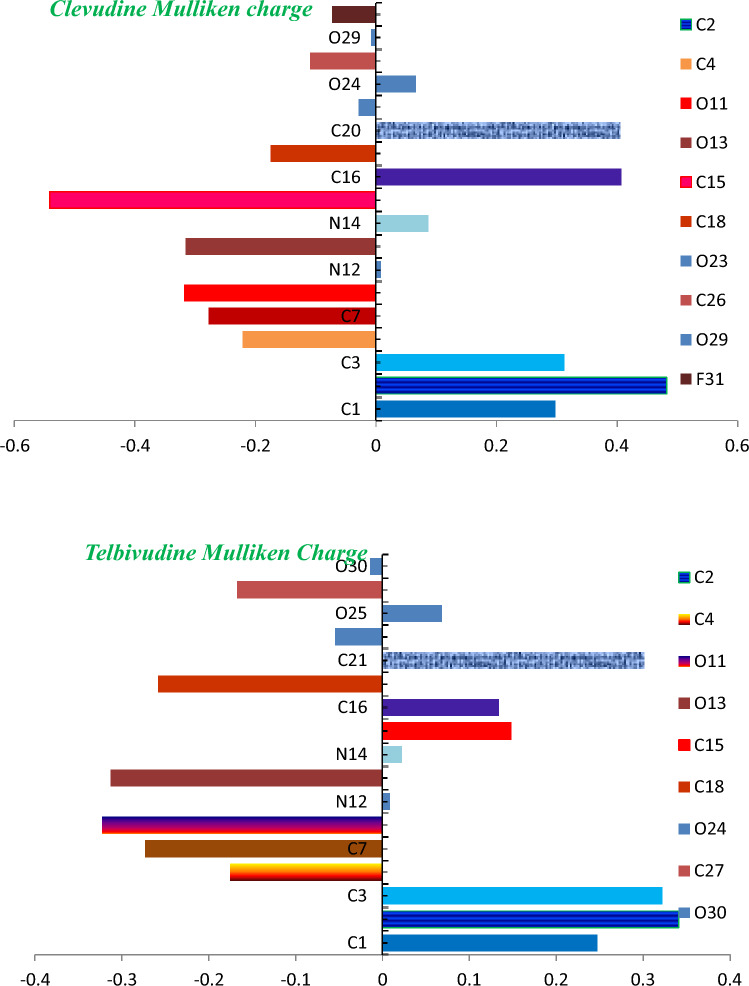
Clevudine and Telbivudine’s Mulliken charges on hydrogens summed to heavy atoms.

Figure 6 displays the Mulliken atomic charges for the compounds clevudine and telbivudine. C4, C7, O13, O11, C18, and F31 were found to have the most negative atomic charges in clevudine, while C1, C2, C3, C16, and C20 had the highest positive atomic charges. It was discovered that telbivudine had the highest electrophilic susceptibility at locations C4, C7, O11, O13, C18, and C27 because they have the most negative charges. On the other hand, the C1, C2, C3, C15, C16, and C21 sites of telbivudine are vulnerable to nucleophiles. Positively charged centers are the regions that are most susceptible to nucleophilic attacks or electron donation. However, the most negatively charged centers are most likely to bind to the electrophilic ones’ sites. Usually, the interaction of carbon atoms with more electronegative atoms like F, O, and N leads to negatively charged carbon atoms.

### UV spectral analysis

**Figure 7:**
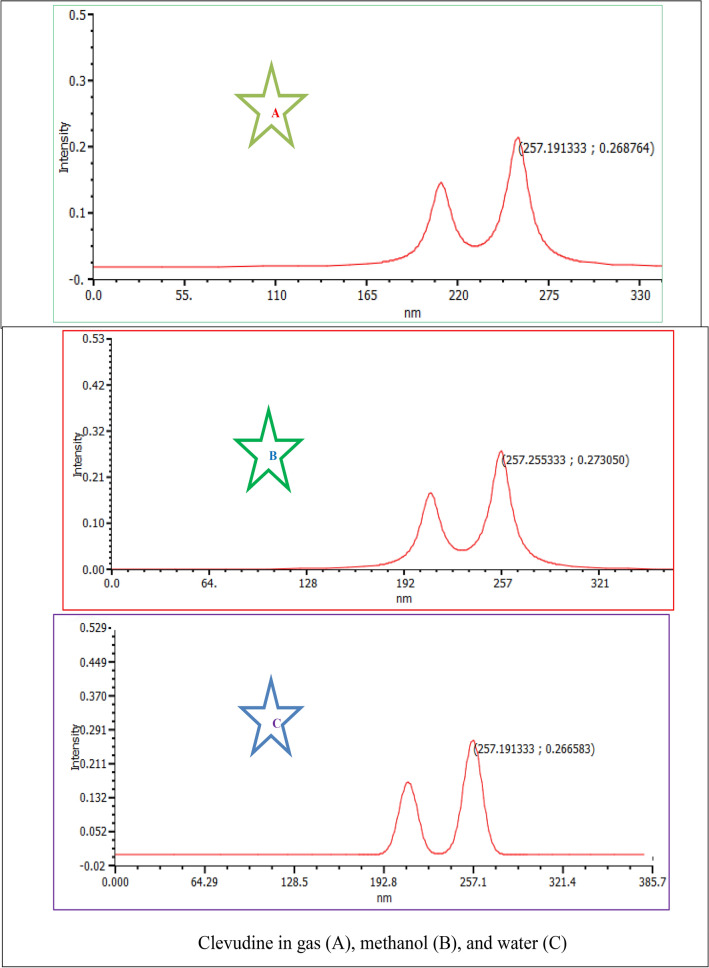

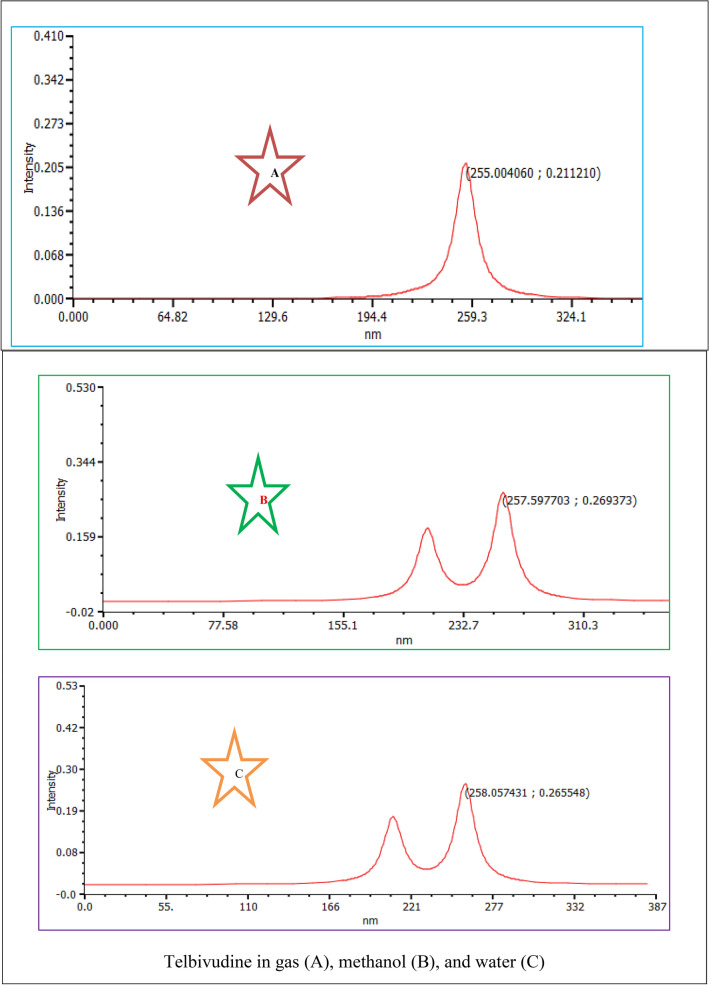
Simulated UV spectra of Clevudine and Telbivudine in the gas phase, methanol, and water solvent.

The TD-DFT computation was used to examine the characteristics of electronic absorption using the lowest singlet spin-allowed excited states of clevudine and telbivudine. The UV absorption spectral calculations were performed utilizing the equilibrium structural characteristics of molecules and TD-DFT with the 6-311++G(d,p) basis set^36–40^. The UV spectra taken in the gas phase, methanol, and water solvents are used to calculate the theoretical “maximum wavelength” values. Table 3 presents the TD-DFT calculations’ results for the first six transitions of the investigated substances’ absorption wavelength, excitation energy (E), and oscillator strength (f). In the electronic absorption spectra of clevudine and telbivudine, there are absorption bands with a maximum wavelength (λ_max_) of 257.19 nm and 259.31 nm, respectively, in the gas phase. In methanol solvent, λ_max_ of Clevudine and Telbivudine is observed at 257.25 nm and 258.61 nm, respectively. However, λ_max_ of Clevudine and Telbivudine is observed at 257.19 nm and 258.65 nm, respectively, in a water solvent. When the medium is changed, Clevudine and Telbivudine have no further impact on the maximum wavelength effect. The *n*–π∗ transitions are responsible for the strong absorption band, and π–π∗ transitions are responsible for the other moderately intense bands. Because of the prolonged aromaticity of the benzene ring, the π–π∗ transitions are predicted to occur substantially at lower wavelengths. The simulated UV spectra of cevudine and telbivudine in the gas phase, methanol, and water solvent are shown in Fig. 7.

**Table 3:** Energies (eV), λ (nm), oscillator strengths (*f*), and transition character of **1** and **2** compounds calculated at TDDFT/B3LYP/6-311++G(d, p) level in gas, methanol, and water.

	Energy (eV)	Wavelength (nm)	Oscillator strength	Transition
Gas phase				
** 1**	4.8207	257.19	0.2647	H $\to$ L
	5.0276	246.61	0.0081	H-4 → L
	5.7716	214.82	0.0017	H → L + 1
	5.7833	214.38	0.0001	H-2 → L
	5.8854	210.66	0.1656	H → L + 2
	6.0030	206.54	0.0035	H → L + 3
** 2**	4.7814	259.31	0.0090	H-1 → L
	4.8598	255.12	0.2052	H → L
	5.2069	238.11	0.0025	H → L + 1
	5.5773	222.30	0.0028	H → L + 2
	5.6426	219.73	0.0006	H → L + 3
	5.8295	212.69	0.0009	H-3 → L
Methanol				
** 1**	4.8195	257.25	0.2675	H $\to$ L
	5.0178	247.09	0.0087	H-1 → L
	5.7679	214.96	0.0019	H → L + 3
	5.7888	214.18	0.0001	H-2 → L
	5.8835	210.73	0.1664	H → L + 2
	5.9800	207.33	0.0041	H → L + 1
** 2**	4.7942	258.61	0.2653	H → L
	5.0310	246.44	0.0031	H-1 → L
	5.6608	219.02	0.0024	H → L + 1
	5.7391	216.03	0.0019	H-2 → L
	5.9175	209.52	0.1724	H → L + 2
	5.9926	206.90	0.0010	H → L + 3
Water				
** 1**	4.8207	257.19	0.2647	H $\to$ L
	5.0276	246.61	0.0081	H-1 → L
	5.7716	214.82	0.0017	H → L + 1
	5.7833	214.38	0.0001	H → L + 2
	5.8854	210.66	0.1656	H → L + 1
	6.0030	206.54	0.0035	H-4 → L
** 2**	4.7935	258.65	0.2661	H → L
	5.0387	246.06	0.0029	H-1 → L
	5.6657	218.83	0.0024	H → L + 1
	5.7352	216.18	0.0020	H-2 → L
	5.9160	209.57	0.1732	H → L + 2
	6.0038	206.51	0.0016	H → L + 3

### Infra-red spectra analysis

**Figure 8:**
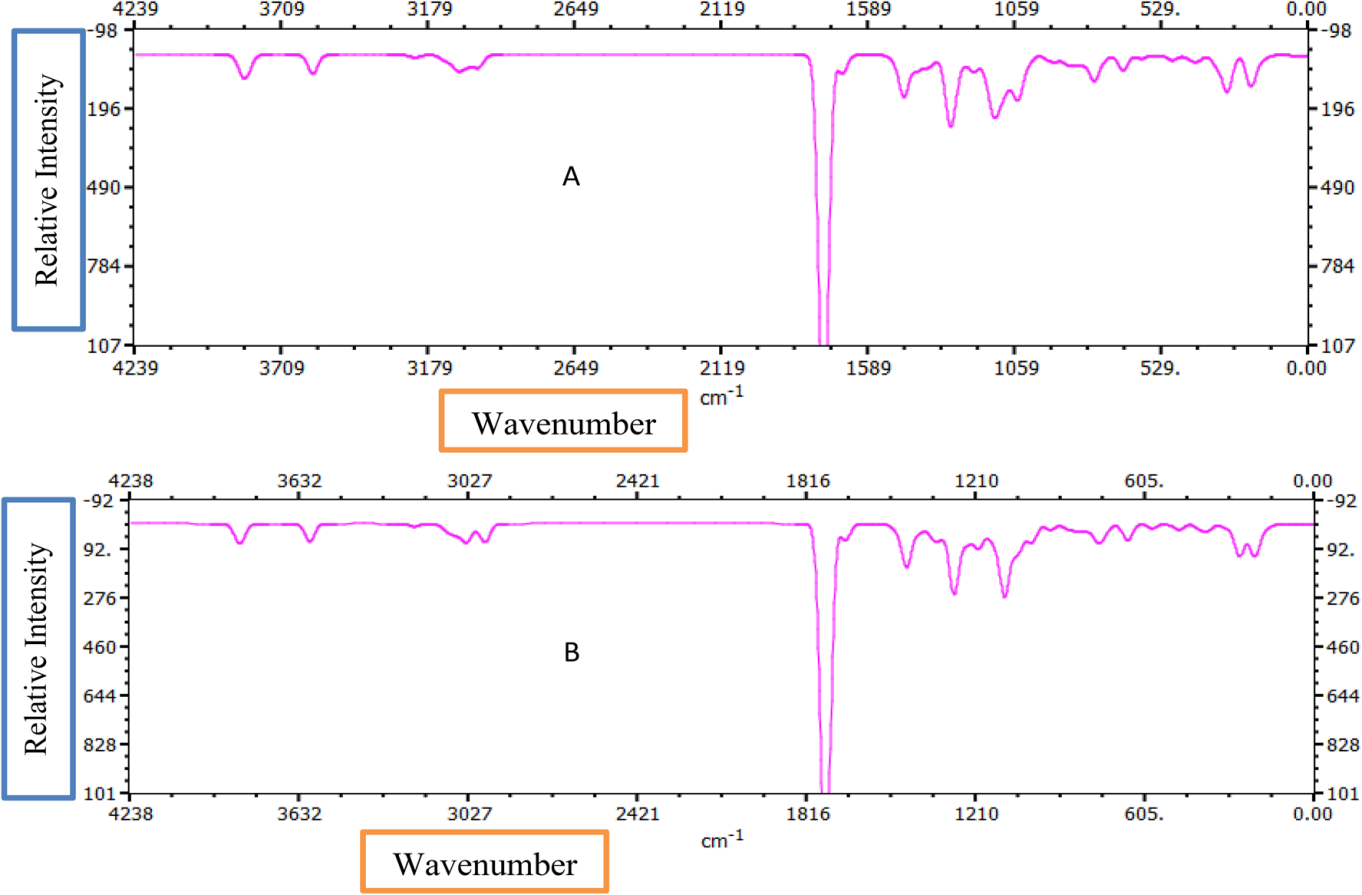
Simulated IR Spectrum of Clevudine (1) and Telbivudine (2).

The structural properties of the studied clevudine and telbivudine compounds’ more stable conformers were used to determine the IR vibrational wavenumbers. Each of the 31 atoms that make up telbivudine and clevudine has 87 active vibrational modes. Based on the collected data, it is predicted that the C–H and O–H stretching modes will be observed in the high wavenumber region. The C–H stretching modes of the methylene groups and aromatic rings were identified. The C–H stretching bands of Clevudine are observed between 3032.32 and 3227.12 cm^−1^ in the IR spectrum. The C-H stretching bands of telbivudine are observed between 3031.38 and 3220.64 cm^−1^ in the IR spectrum. Figure 8 shows the simulated IR spectrum of Clevudine and Telbivudine. For Clevudine and Telbivudine, the O–H stretching mode is observed around 3833.6 cm^−1^ and 3836.7 cm^−1^. For Clevudine, the CH_2_ scissoring and wagging modes were also identified. The CH_2_ wagging and scissoring modes are observed at around 1268 cm^−1^ and 1498 cm^−1^, respectively. For Telbivudine, the CH_2_ wagging and scissoring modes are observed at around 1280 cm^−1^ and 1503 cm^-1^, respectively. Clevudine and Telbivudine's scaled N–H stretching vibrations are 3596 cm^−1^ and 3595.8 cm^−1^, respectively.

### Natural bond orbital (NBO) analysis

It is ideal to employ the natural bond orbital (NBO) analysis, which provides details on intra- and intermolecular interactions, to examine the charge transfer in a system between donor and acceptor NBOs^20^. The second-order perturbation energy E^(2)^ expresses the donor (*i*) to acceptor (*j*) interaction as a delocalization of i → j:

$$ {\text{E}}^{{({2})}} = {\text{ q}}_{{\text{i}}} {\text{F}}^{{2}} \left( {{\text{i}},{\text{j}}} \right)/\varepsilon_{{\text{j}}} - \varepsilon_{{\text{i}}} $$

In this equation, q_i_, F (i,j), and ε are the electronic occupancy in the donor orbital, the diagonal element in the orbital energies, and the off-diagonal NBO Fock matrix element, in that order^30–35^. The examination of occupied and unoccupied NBO according to their second-order perturbation energies can help explain the stability (E^(2)^) that shows how an electron can be transferred from a donor NBO to an acceptor one^21^. Calculated delocalization energies for significant intramolecular interactions of molecules like clevudine and telbivudine. As can be seen from Table 4, the most remarkable interaction for Clevudine is attributed to the BD*(2) C4-O11 → BD*(2) C2-C3, which has resonance energy equal to 114.80 kcal mol^−1^. Moreover, the LP (1) N12 → BD*(2) C1-O13 and LP (1) N14 → BD*(2) C1-O13 transition shows another significant interaction with 60.58 kcal mol^−1^ and 56.61 kcal mol^−1^, respectively. Such interactions were a result of aromaticity and pi-bond delocalization, which contribute significantly to ring current and stability. Resonance energy for the delocalization of the electron of BD (1) C2-C3 → BD*(2) C4-O11 is 22.99 kcal mol^−1^, indicating that the C2-C3 and C4-O11 bonds can be conjugated easily. These results demonstrate that the aromaticity and conjugation of the clevudine molecules are related to their strongest interactions. According to Table 5, the most remarkable interaction for Telbivudine is attributed to the BD*(2) C4-O11 → BD*(2) C2-C3, which has resonance energy equal to 138.50 kcal mol^−1^. Moreover, the LP (1) N12 → BD*(2) C1-O13 and LP (1) N14 → BD*(2) C1-O13 transition shows another significant interaction with 59.64 kcal mol^−1^ and 57.85 kcal mol^−1^ respectively. Such interactions were a result of aromaticity and bond delocalization, which contribute significantly to ring current and stability. Resonance energy for the electron delocalization of BD (1) C2-C3 → BD*(2) C4-O11 is 22.97 kcal mol^−1^, indicating that the C2-C3 and C4-O11 bonds can be conjugated easily. These results demonstrate that the aromaticity and conjugation of the telbivudine molecule are related to the strongest interactions in the molecule.

**Table 4:** Significantly calculated donor–acceptor interactions in Clevudine at B3LYP/6-311++G (d, p) level of theory.

Donor NBO (i)	Acceptor NBO (j)	E(2) (kcal mol^−1^)	E(j)−E(i) (a.u.)	F(i,j) (a.u.)
BD (1) C1-N12	BD*(1) C1-O13	1.42	1.43	0.040
BD (1) C1-N12	BD*(1) C4-O11	1.58	1.46	0.043
BD (1) C1-N12	BD*(1) C4-N12	0.78	1.25	0.028
BD (1) C1-N12	BD*(1) N14-C15	2.43	1.15	0.048
BD (1) C1-O13	BD*(1) C1-N12	1.66	1.49	0.045
BD (1) C1-O13	BD*(1) C1-N14	1.25	1.47	0.039
BD (1) C1-O13	BD*(1) C2-N14	1.73	1.49	0.046
BD (1) C1-O13	BD*(1) C4-N12	1.38	1.47	0.041
BD (2) C1-O13	BD*(2) C1-O13	2.20	0.38	0.029
BD (1) C1-N14	BD*(2) C1-O13	1.17	1.41	0.036
BD (1) C1-N14	BD*(1) C2-H6	1.35	1.26	0.037
BD (1) C1-N14	BD*(1) C2-N14	1.95	1.26	0.044
BD (1) C1-N14	BD*(1) H5-N12	1.80	1.21	0.042
BD (1) C1-N14	BD*(1) N14-C15	0.87	1.13	0.028
BD (1) C1-N14	BD*(1) C15-O23	1.23	1.11	0.033
BD (1) C2-C3	BD*(1) C2-H6	1.81	1.19	0.041
BD (1) C2-C3	BD*(1) C2-N14	2.06	1.18	0.044
BD (1) C2-C3	BD*(1) C3-C4	1.88	1.23	0.043
BD (1) C2-C3	BD*(1) C3-C7	2.67	1.18	0.050
BD (1) C2-C3	BD*(1) C4-O11	2.51	1.37	0.052
BD (1) C2-C3	BD*(1) N14-C15	3.04	1.05	0.051
BD (1) C2-C3	BD*(2) C2-C3	2.35	0.31	0.024
BD (1) C2-C3	BD*(2) C4-O11	22.99	0.30	0.077
BD (1) C2-C3	BD*(1) C7-H8	2.97	0.70	0.042
BD (1) C2-C3	BD*(1) C7-H9	2.95	0.70	0.042
BD (1) C2-H6	BD*(1) C1-N14	4.60	0.95	0.060
BD (1) C2-H6	BD*(1) C2-C3	2.33	1.18	0.047
BD (1) C2-H6	BD*(1) C3-C4	4.41	1.02	0.060
BD (1) C2-N14	BD*(1) C1-O13	2.35	1.42	0.052
BD (1) C2-N14	BD*(1) C1-N14	1.40	1.23	0.038
BD (1) C2-N14	BD*(1) C2-C3	1.99	1.46	0.048
BD (1) C2-N14	BD*(1) C3-C7	2.63	1.26	0.051
BD (1) C2-N14	BD*(1) N14-C15	1.45	1.13	0.036
BD (1) C3-C4	BD*(1) C2-C3	2.52	1.31	0.051
BD (1) C3-C4	BD*(1) C2-H6	3.29	1.10	0.054
BD (1) C3-C4	BD*(1) C3-C7	0.94	1.10	0.029
BD (1) C3-C4	BD*(1) C4-O11	2.27	1.28	0.048
BD (1) C3-C4	BD*(1) H5-N12	2.50	1.06	0.046
BD (1) C3-C4	BD*(1) C7-H10	0.92	1.09	0.028
BD (1) C3-C7	BD*(1) C2-C3	3.64	1.26	0.061
BD (1) C3-C7	BD*(1) C 2-N14	4.15	1.06	0.059
BD (1) C3-C7	BD*(1) C3-C4	1.23	1.10	0.033
BD (1) C3-C7	BD*(1) C4-N12	2.34	1.03	0.045
BD (1) C3-C7	BD*(1) C7-H8	0.51	1.05	0.021
BD (1) C4-O11	BD*(1) C1-N12	1.33	1.48	0.040
BD (1) C4-O11	BD*(1) C2-C3	0.79	1.69	0.033
BD (1) C4-O11	BD*(1) C3-C4	2.49	1.53	0.056
BD (1) C4-O11	BD*(1) C4-N12	0.70	1.46	0.029
BD (2) C4-O11	BD*(2) C2-C3	4.95	0.40	0.042
BD (2) C4-O11	BD*(2) C4-O11	0.85	0.38	0.017
BD (1) C4-N12	BD*(1) C1-N12	0.75	1.24	0.028
BD (1) C4-N12	BD*(1) C1-O13	2.46	1.40	0.052
BD (1) C4-N12	BD*(1) C3-C7	2.18	1.24	0.046
BD (1) C4-N12	BD*(1) C4-O11	0.56	1.42	0.025
BD (1) H5-N12	BD*(1) C1-N14	3.60	1.07	0.056
BD (1) H5-N12	BD*(1) C3-C4	2.38	1.14	0.047
BD (1) H5-N12	BD*(1) C4-O11	0.64	1.28	0.026
BD (1) C7-H8	BD*(1) C2-C3	2.11	1.12	0.044
BD (1) C7-H8	BD*(2) C2-C3	3.63	0.52	0.041
BD (1) C7-H9	BD*(1) C2-C3	2.14	1.12	0.044
BD (1) C7-H9	BD*(2) C2-C3	3.60	0.52	0.040
BD (1) C7-H10	BD*(1) C3-C4	4.12	0.97	0.057
LP (1) O11	BD*(1) C3-C4	2.39	1.15	0.047
LP (1) O11	RY*(1) C4	16.69	1.78	0.154
LP (2) O11	BD*(1) C3-C4	17.06	0.72	0.101
LP (2) O11	BD*(1) C4-N12	28.62	0.65	0.123
LP (1) N12	BD*(2) C1-O13	60.58	0.27	0.115
LP (1) N12	BD*(2) C4-O11	47.53	0.29	0.106
LP (1) O13	BD*(1) C1-N12	1.71	1.13	0.040
LP (1) O13	BD*(1) C1-N14	1.54	1.10	0.037
LP (2) O13	BD*(1) C1-N12	24.22	0.69	0.117
LP (2) O13	BD*(1) C1-N14	25.59	0.66	0.118
LP (2) O13	BD*(1) C15-O23	1.10	0.55	0.023
LP (1) N14	BD*(2) C1-O13	56.61	0.28	0.112
LP (1) N14	BD*(2) C2-C3	35.60	0.31	0.099
LP (1) N14	BD*(1) C15-C16	6.43	0.60	0.060
LP (1) N14	BD*(1) C15-H17	2.74	0.67	0.042
LP (1) N14	BD*(1) C15-O23	1.06	0.56	0.024
LP (1) O23	BD*(1) N14-C15	0.79	0.88	0.024
LP (1) O23	BD*(1) C15-C16	2.82	0.90	0.045
LP (1) O23	BD*(1) C15-H17	0.60	0.97	0.022
LP (1) O23	BD*(1) C18-C20	1.89	0.92	0.037
LP (1) O23	BD*(1) C20-C26	0.72	0.96	0.024
BD*(2) C4-O11	BD*(2) C2-C3	114.80	0.02	0.076

**Table 5:** Significantly calculated donor–acceptor interactions in Telbivudine at B3LYP/6-311++G (d, p) level of theory.

Donor NBO (i)	Acceptor NBO (j)	E(2) (kcal mol^−1^)	E(j)–E(i) (a.u.)	F(i,j) (a.u.)
BD (1) C1-N12	BD*(1) C1-O13	1.37	1.43	0.040
BD (1) C1-N12	BD*(1) C4-O11	1.63	1.45	0.043
BD (1) C1-N12	BD*(1) C4-N12	0.77	1.24	0.028
BD (1) C1-N12	BD*(1) N14-C15	2.39	1.12	0.047
BD (1) C1-O13	BD*(1) C1-N12	1.61	1.49	0.044
BD (1) C1-O13	BD*(1) C1-N14	1.32	1.47	0.040
BD (1) C1-O13	BD*(1) C2-N14	1.77	1.50	0.046
BD (1) C1-O13	BD*(1) C4-N12	1.38	1.47	0.041
BD (2) C1-O13	BD*(2) C1-O13	2.20	0.38	0.029
BD (1) C1-N14	BD*(2) C1-O13	1.22	1.42	0.037
BD (1) C1-N14	BD*(1) C2-H6	1.41	1.26	0.038
BD (1) C1-N14	BD*(1) C2-N14	1.90	1.26	0.044
BD (1) C1-N14	BD*(1) H5-N12	1.77	1.21	0.041
BD (1) C1-N14	BD*(1) N14-C15	0.69	1.11	0.025
BD (1) C1-N14	BD*(1) C15-O24	1.06	1.12	0.031
BD (1) C2-C3	BD*(1) C2-H6	1.82	1.18	0.041
BD (1) C2-C3	BD*(1) C2-N14	2.14	1.19	0.045
BD (1) C2-C3	BD*(1) C3-C4	1.86	1.23	0.043
BD (1) C2-C3	BD*(1) C3-C7	2.65	1.18	0.050
BD (1) C2-C3	BD*(1) C4-O11	2.54	1.36	0.053
BD (1) C2-C3	BD*(1) N14-C15	3.04	1.03	0.051
BD (1) C2-C3	BD*(2) C2-C3	2.48	0.31	0.025
BD (1) C2-C3	BD*(2) C4-O11	22.97	0.30	0.077
BD (1) C2-C3	BD*(1) C7-H8	2.97	0.70	0.042
BD (1) C2-C3	BD*(1) C7-H9	2.98	0.70	0.042
BD (1) C2-H6	BD*(1) C1-N14	4.64	0.95	0.060
BD (1) C2-H6	BD*(1) C2-C3	2.25	1.18	0.046
BD (1) C2-H6	BD*(1) C3-C4	4.41	1.02	0.060
BD (1) C2-N14	BD*(1) C1-O13	2.39	1.42	0.052
BD (1) C2-N14	BD*(1) C1-N14	1.34	1.24	0.037
BD (1) C2-N14	BD*(1) C2-C3	2.05	1.46	0.049
BD (1) C2-N14	BD*(1) C3-C7	2.62	1.26	0.051
BD (1) C2-N14	BD*(1) N14-C15	1.21	1.11	0.033
BD (1) C3-C4	BD*(1) C2-C3	2.50	1.30	0.051
BD (1) C3-C4	BD*(1) C2-H6	3.29	1.10	0.054
BD (1) C3-C4	BD*(1) C3-C7	0.94	1.10	0.029
BD (1) C3-C4	BD*(1) C4-O11	2.26	1.28	0.048
BD (1) C3-C4	BD*(1) H5-N12	2.53	1.06	0.046
BD (1) C3-C4	BD*(1) C7-H10	0.92	1.09	0.028
BD (1) C3-C7	BD*(1) C2-C3	3.60	1.26	0.060
BD (1) C3-C7	BD*(1) C2-N14	4.10	1.06	0.059
BD (1) C3-C7	BD*(1) C3-C4	1.23	1.10	0.033
BD (1) C3-C7	BD*(1) C4-N12	2.32	1.03	0.044
BD (1) C3-C7	BD*(1) C7-H8	0.51	1.05	0.021
BD (1) C4-O11	BD*(1) C1-N12	1.35	1.48	0.041
BD (1) C4-O11	BD*(1) C2-C3	0.79	1.69	0.033
BD (1) C4-O11	BD*(1) C3-C4	2.49	1.53	0.056
BD (1) C4-O11	BD*(1) C4-N12	0.72	1.46	0.030
BD (2) C4-O11	BD*(2) C2-C3	4.94	0.40	0.041
BD (2) C4-O11	BD*(2) C4-O11	0.89	0.38	0.018
BD (1) C4-N12	BD*(1) C1-N12	0.73	1.23	0.027
BD (1) C4-N12	BD*(1) C1-O13	2.40	1.40	0.052
BD (1) C4-N12	BD*(1) C3-C7	2.20	1.24	0.047
BD (1) C4-N12	BD*(1) C4-O11	0.57	1.42	0.026
BD (1) H5-N12	BD*(1) C1-N14	3.53	1.07	0.056
BD (1) H5-N12	BD*(1) C3-C4	2.41	1.14	0.047
BD (1) H5-N12	BD*(1) C4-O11	0.64	1.28	0.025
BD (1) C7-H8	BD*(1) C2-C3	2.14	1.12	0.044
BD (1) C7-H8	BD*(2) C2-C3	3.58	0.52	0.040
BD (1) C7-H9	BD*(1) C2-C3	2.13	1.12	0.044
BD (1) C7-H9	BD*(2) C2-C3	3.60	0.52	0.040
BD (1) C7-H10	BD*(1) C3-C4	4.11	0.97	0.057
LP (1) O11	BD*(1) C3-C4	2.41	1.15	0.048
LP (1) O11	RY*(1) C4	16.69	1.78	0.154
LP (2) O11	BD*(1) C3-C4	17.01	0.72	0.101
LP (2) O11	BD*(1) C4-N12	28.44	0.65	0.123
LP (1) N12	BD*(2) C1-O13	59.64	0.27	0.114
LP (1) N12	BD*(2) C4-O11	48.38	0.29	0.107
LP (1) O13	BD*(1) C1-N12	1.68	1.12	0.039
LP (1) O13	BD*(1) C1-N14	1.55	1.10	0.037
LP (2) O13	BD*(1) C1-N12	24.62	0.68	0.118
LP (2) O13	BD*(1) C1-N14	25.28	0.67	0.118
LP (2) O13	BD*(1) C15-O24	1.06	0.55	0.022
LP (1) N14	BD*(2) C1-O13	57.85	0.27	0.113
LP (1) N14	BD*(2) C2-C3	37.32	0.31	0.100
LP (1) N14	BD*(1) C15-C16	4.19	0.61	0.050
LP (1) N14	BD*(1) C15-H17	3.48	0.68	0.048
LP (1) N14	BD*(1) C15-O24	1.06	0.55	0.022
LP (1) O24	BD*(1) N14-C15	11.39	0.60	0.074
LP (1) O24	BD*(1) C15-C16	2.95	0.92	0.046
LP (1) O24	BD*(1) C15-H17	3.48	0.68	0.048
LP (1) O24	BD*(1) C18-C20	1.44	0.93	0.033
LP (1) O24	BD*(1) C20-C26	0.96	0.95	0.027
BD*(2) C4-O11	BD*(2) C2-C3	138.50	0.01	0.076

## Conclusion

In this study, electronic structural analysis, energy of Highest occupied molecular orbital (HOMO), and energy of Lowest unoccupied molecular orbital (LUMO), (E_gap_), global reactivity descriptors, Dipole moment, molecular electrostatic potential profile, Mulliken charge analysis, natural bond orbital, IR and UV spectra of Clevudine and Telbivudine were calculated using the DFT/TDDFT method with B3LYP/6-311++G (d, p) level of theory. From the calculated data, Clevudine and Telbivudine have a non-planar structure, and the Energy Gap of clevudine is 4.1653 eV and Telbivudine is 6.6865 eV. The DFT estimated data revealed that the dipole moment of the compounds under study is in the order of clevudine < telbivudine. The high dipole moment shows their binding pose within a specific target protein and the results of the predicted binding affinity. For Clevudine and Telbivudine compounds, the electrophilic and nucleophilic region lies in the C=O group of the pyrimidine ring. In NBO, most of the strong interactions in clevudine and telbivudine compounds are related to aromaticity and conjugation. NBO analysis provides electronic insights, UV–Vis reveals electronic transitions, and IR confirms the molecular structure. These techniques play a vital role in drug design, including potential treatments for Hepatitis B.

## Data Availability

The datasets generated during and/or analyzed during the current study are not publicly available but are available from the corresponding author on reasonable request.

## Abbreviations

EA: Electron affinity
EN: Electronegativity
DFT: Density functional theory
TD-DFT: Time-dependent density functional theory
DNA: Deoxyribonucleic acid
E: Excitation energy
FMOs: Frontier molecular orbitals
HOMO: Highest occupied molecular orbital
LUMO: Lowest unoccupied molecular orbital
IR: Infrared
MEP: Molecular electrostatic potential
NBO: Natural bond orbital
N: Nucleophilicity index
IP: Ionization potential
UV: Ultraviolet
